# Editorial: Advances and trends in gene expression, regulation, and phenotypic variation in livestock science: a comprehensive review of methods and technologies

**DOI:** 10.3389/fgene.2025.1565301

**Published:** 2025-02-13

**Authors:** Priyanka Banerjee, Andressa Oliveira de Lima, Loan T. Nguyen, Wellison J. S. Diniz

**Affiliations:** ^1^ Department of Animal Sciences, Auburn University, Auburn, AL, United States; ^2^ Department of Genome Sciences, University of Washington, Seattle, WA, United States; ^3^ Queensland Alliance for Agriculture and Food Innovation, University of Queensland, St Lucia, QLD, Australia

**Keywords:** computational genomics, epigenetics, genetic variants, genomics, livestock

Breeding for efficient animals has been a major focus for producers, aiming not only to increase profitability but also to address the societal demands for sustainability. Advances in next-generation sequencing over the last decades have revolutionized our understanding of the regulatory mechanisms related to animal health and production. These advances, coupled with new analytical methods, are helping to bridge the genome-to-phenome gap, yielding positive impacts on selective breeding (Clark et al., 2020). Furthermore, genome editing through the CRISPR-cas9 system has been a paradigm shift, offering new opportunities to introduce genetic variants with the potential to maximize animal production (Banerjee and Diniz, 2024; Mueller and Van Eenennaam, 2022).

In the current Research Topic (RT), we collected reviews, case reports, and original research articles from experts, highlighting the progress of high-throughput technologies and their applications to livestock science. Our objective is to provide an overview of the recent genomic technologies and enhance our understanding of gene regulation, epigenetics, genome architecture, and its crosstalk with phenotypic variation underlying animal production, nutrition, reproduction, health, and environmental adaptation. This RT includes five scientific articles spanning Research Topic from genome to epigenome, including nutrigenomics and metabolomics in various species.

The article by Chen et al. provided a comprehensive overview of technologies, methods, and applications to study genomic structural variants (SV). They discussed the mechanisms of SV formation and presented the evolution of methods to detect structural variants. Moreover, they reviewed studies across multiple species (cattle, buffalo, equine, sheep, and goats) to elucidate the genetic basis of differences in phenotypic traits and adaptive genetic mechanisms linked to SVs. The authors highlighted the significance of SVs as a source of genetic diversity among individuals. However, they pointed out the need for further development of efficient and cost-effective long-read sequencing technologies and improved algorithms designed to manage the complexity of genomic regions. Taking advantage of public data and whole-genome resequencing, the research article by Liu et al. investigated the genomic architecture of Xiangdong black goat compared to six other goat breeds (Jintang, Jining, Guizhou, Du’an, Chengdu, and Longlin) in China. They analyzed population structure, genome diversity, and selective signatures. Among their findings, they reported a moderate level of diversity in Xiangdong goats, minimal inbreeding, and a substantial effective population size. The selective sweep analysis also identified candidate genes involved in immunity, reproduction, and growth and development.

Two other research articles focused on functional genomics, uncovering potential regulatory mechanisms in pigs. Liu et al. explored the regulatory dynamics underlying phenotypic divergence in pigs by analyzing cis-regulatory elements (CREs). The authors compared chromatin accessibility profiles in skeletal muscle, liver, and fat tissues between Chinese (Meishan and Enshi Black) and Western (Duroc and Large White) pig populations. They also explored whether the chromatin accessibility changes were underlying changes in liver gene expression. Their findings highlighted liver-specific regulatory elements, with increased chromatin accessibility in Chinese pigs correlating with higher expression levels of nearby genes. Another study by Fanalli et al. examined the dietary modulation of liver gene expression to understand animal health and production. Using RNA-Seq and co-expression network analyses, the study examined the impact of diets containing 3% soybean oil (SOY3.0) versus a standard 1.5% soybean oil diet (SOY1.5) on blood serum parameters and liver gene expression in 98-day-old pigs. The authors reported distinct co-expression modules: co-expressed genes from the SOY3.0 treatment were associated with neurodegeneration, Alzheimer’s disease, and metabolic processes pathways, while SOY1.5 modulated pathways were related to cholesterol metabolism and type I diabetes mellitus. These results highlight the potential of nutrigenomics to develop dietary strategies for optimizing livestock metabolic health. Moreover, this research illustrates the translational relevance of livestock studies for human health by offering insights into how tailored fatty acid profiles could enhance human lipid metabolism and reduce disease risks, including cardiovascular and neurodegenerative disorders.

Finally, advancing from livestock to companion animals, Katz et al. reported on a pioneering application of metabolic gene therapy for treating pulmonary hypertension (PH) in dogs. The authors reviewed the prevalence and pathophysiology of PH, and demonstrated that synthetic adeno-associated virus-mediated gene delivery of acid ceramidase has the potential to significantly reduce the need for drug treatment and improve spontaneously occurring PH in dogs.

Collectively, the contributions within this RT showcase the transformative potential of genomic technologies, from elucidating structural variation to uncovering genome regulation. These findings provide a foundation for future research to bridge the gaps in our understanding of complex traits, advancing the sustainability and efficiency of animal production systems.
